# Modelling the Inactivation and Possible Regrowth of *Salmonella enterica* Treated with Chlorophyllin-Chitosan Complex and Visible Light

**DOI:** 10.17113/ftb.58.01.20.6374

**Published:** 2020-03

**Authors:** María Isabel Rodríguez-López, Vicente M. Gómez-López, Viktorija Lukseviciute, Zivile Luksiene

**Affiliations:** 1 Departamento de Ciencia y Tecnología de Alimentos, Universidad Católica de Murcia (UCAM), Campus de los Jerónimos 135, 30107 Guadalupe, Murcia, Spain; 2 Cátedra Alimentos para la Salud, Universidad Católica de Murcia (UCAM), Campus de los Jerónimos 135, 30107 Guadalupe, Murcia, Spain; 3 Institute of Computer Science, Vilnius University, Didlaukio g. 47, 08303 Vilnius, Lithuania; 4 Institute of Photonics and Nanotechnology, Vilnius University, Saulėtekio 10, 10223 Vilnius, Lithuania

**Keywords:** photosensitization, microbial modelling, treatment with chlorophyllin and chitosan complex, *Salmonella enterica*, microbial inactivation

## Abstract

The study focuses on predictive modelling of inactivation of *Salmonella enterica* after treatment with chlorophyllin-chitosan complex and visible light. *Salmonella* cells were incubated with chlorophyllin-chitosan complex (0.001% chlorophyllin and 0.1% chitosan) for different times (5-60 min) and then illuminated with visible light (*λ*=405 nm, *H*_e_=38 J/cm^2^). Inactivation curves and post-treatment regrowth curves were built based on microbiological viability tests and data were fitted to ten inactivation and two regrowth models. The photoactivated complex reduced *Salmonella* population, which were unable to regrow. Weibull and Baranyi models were the best to describe the inactivation and regrowth kinetics respectively. In conclusion, data from the kinetic analysis and predictive modelling confirmed that photoactivated chlorophyllin-chitosan complex is a promising non-thermal approach for inactivation of Gram-negative pathogens, since no bacterial regrowth after treatment has been predicted.

## INTRODUCTION

According to World Health Organization (WHO) the incidence of foodborne diseases is a drastically growing public health problem in the world (*1*). Likewise, the Centers for Disease Control and Prevention (CDC) reported 48 million illnesses, 128 000 hospitalizations and 3000 deaths every year due to foodborne illness caused by pathogenic microorganisms (*2*). Fresh produce has become the second leading cause of foodborne illnesses, which poses a US$77.7 billion economic burden in the US annually (*3*). *Salmonella* is one of the most important foodborne pathogens. In 2013, *Salmonella* affected more than one million people in the USA, with 19 336 hospitalizations and 378 deaths, and associated costs over US$3.7 billion (*4*). A recent study collected outbreak data from 2007 to 2011 in the European Union has identified *Salmonella* in ready-to-eat unprocessed foods of non-animal origin as the microorganism most often linked to human cases of foodborne illnesses (*5*). Consequently, the control of foodborne infections remains a global problem with significant social and economic impact (*6*). In this context, innovative, effective, non-chemical and environmentally friendly antimicrobial technologies are in high demand.

To this end, a modern biophotonic technology based on photosensitization and successfully used to cure cancer and infectious diseases (photodynamic therapy) (*7*) is under study for the decontamination of fresh produce and food-related surfaces (*8*-*10*). It is interesting to note that chlorophyllin (E140ii), which is permitted for use as a food colourant in the European Union in accordance with Annex II to Regulation (EC) No 1333/2008 (*11*) and in the USA according to regulation 21CFR73.125 (*12*), can act as very effective photosensitizer (*13*). This nonthermal treatment is based on the combined action of photosensitizer, light and oxygen, which eventually produces reactive oxygen species and triggers the death of all microorganisms that interact with the photosensitizer (*13*). At molecular level, photoactivated chlorophyllin inactivates bacteria by generation of singlet oxygen (^1^O_2_), causing oxidative stress and increasing cell membrane permeability, which occurs while bacterial cell upregulates genes responsible for detoxification of reactive oxygen species and downregulates genes responsible for inhibition of oxidative respiration, cell division and metabolism (*14*).

The main advantage of photosensitization is its high efficiency against a wide range of microorganisms: Gram-positive and Gram-negative bacteria, their vegetative forms and spores, as well as fungi and yeasts and is as effective as high-power pulsed UV light (*15*). Moreover, this treatment is environmentally friendly, cost-effective, saving water and energy (*16*). However, there is one important disadvantage of this antimicrobial treatment: the susceptibility of Gram-negative bacteria to photosensitization using negatively charged photosensitizer chlorophyllin is lower than that of Gram-positive bacteria (*17*, *18*). This new challenge prompted us to turn to the hurdle technologies, *i.e*. to combine chlorophyllin-based photosensitization with other antimicrobials (*14*).

Chitosan is a food additive derived from chitin, also approved in the USA and European Union (*19*). It can form a chlorophyllin-chitosan complex by interaction between its positively charged NH^3+^ group and the negatively charged chlorophyllin COO¯ group, which can be excited at 405 nm (*8*).

In order to compare quantitatively the efficiencies of different antimicrobial treatments, modelling of the inactivation of bacteria and their regrowth dynamics is most reliable. While modelling of bacterial growth after the treatment is relatively easy (since growth curves have a single exponential or sigmoidal shape), modelling of inactivation curves is not straightforward due to the wide variety of shapes that occur, ranging from a simple log-linear shape to complex multiphasic ones. The latter has given place to several models as summarized by Geeraerd *et al*. (*20*). Usually, the selection of the best fitting model is based on statistical fitting.

The goal of this research is to evaluate, using mathematical models, the inactivation of *Salmonella enterica* treated with photoactivated chlorophyllin-chitosan complex and to predict the possible regrowth of this bacterium after the treatment.

## MATERIALS AND METHODS

### Experiments

**Fig. 1:**
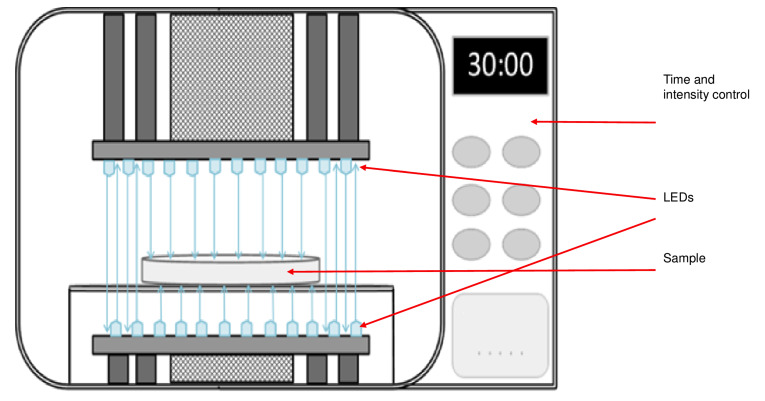
LED-based light source with illumination of the sample from both sides, time and intensity control

All experimental conditions for inactivation of bacteria and data are described by Buchovec *et al*. (*9*, *14*). In brief, experiments were carried out with *Salmonella enterica* serovar Typhimurium strain DS88 [SL5676 SmR (pLM32)] resistant to tetracycline. Chlorophyllin sodium salt without copper (Roth, Karlsruhe, Germany) and low-molecular-mass chitosan (Sigma-Aldrich, Merck, Saint Louis, MO, USA) were used for the preparation of the complex. The complex with 0.001% chlorophyllin and 0.1% chitosan in 0.9% NaCl was used for experiments. For inactivation with light (radiant exposure of 38 J/cm^2^) we used a home-made light device (Fig. 1) equipped with 60 light emitting diodes (LEDs), VIOLET LED emitter (model LZ1-00UA00; LED Engin Inc., San Jose, CA, USA) with peak emission at 405 nm, as described by Buchovec *et al*. (*9*). Microbial inactivation experiments consisted in the incubation of *Salmonella* with chlorophyllin-chitosan complex for 5, 10, 15, 30, 45 or 60 min and subsequent illumination. The samples of 150 µL were placed in sterile flat bottom wells at room temperature and pH=7.2. The treatments were as follows: *i*) control (no sensitizer, no illumination), *ii*) chitosan without illumination, *iii*) photoactivated chlorophyllin, *iv*) chlorophyllin-chitosan complex without illumination, and *v*) photoactivated chlorophyllin-chitosan complex. Then, *S. enterica* surviving populations were enumerated by plate counting. Microbial regrowth was determined in treatments with the complex, photoactivated complex and control by measuring absorbance at 540 nm during incubation in the dark at 37 °C. Experiments were repeated four times.

### Modelling of Salmonella inactivation by photoactivated chlorophyllin-chitosan complex

Inactivation kinetics was analysed using GinaFit add-in tool for Excel (*20*-*22*). Data were fitted to all ten different kinetic models available in this software; only the three models that show the best fit to the data have been included in this report.

The log-linear model reads as follows:
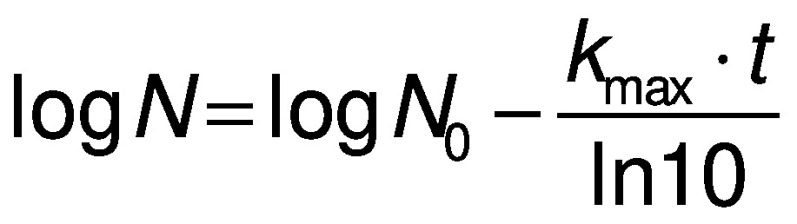
where *N* is the number of microorganisms that survived the different treatments (CFU/mL), *N*_0_ is the initial population (CFU/mL), *k*_max_ is the specific inactivation rate (min^-1^) and *t* the treatment time (min).

The Weibull model reads as follows:
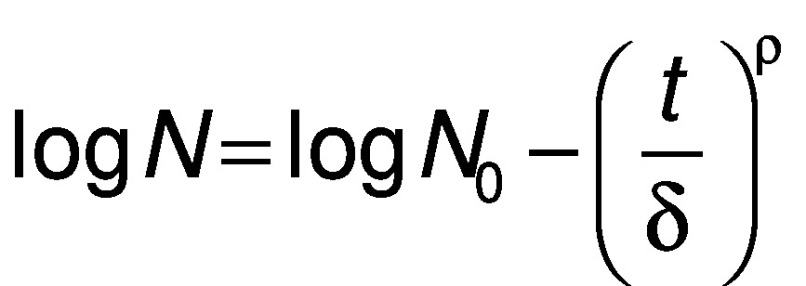
where *δ* is the scale parameter (min) and *ρ* is the shape of the curves (dimensionless). When *ρ*<1, the curve is concave; when *ρ*>1, the curve is convex.

The log-linear and tail model (*20*) read as follows:
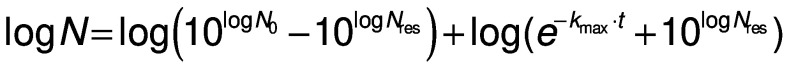
where *N*_res_ (CFU/mL) is the residual population density.

To evaluate the degree of adjustment of the model, the root mean square error (RMSE) and the coefficient of determination R^2^ were used. High R^2^ and low RMSE values indicate a better fit of the model. The RMSE is determined by the following equation:
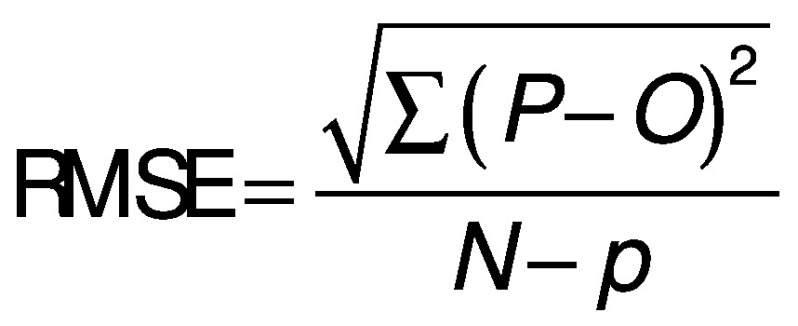
where *P* is the predicted value, *O* the observed value, *N* is the number of observations and *p* is the number of parameters to be estimated.

Weibull model for microbial inactivation was validated with three sets of data from additional tests. Deviations from the predicted values were analysed and the RMSE value was calculated as measurement of the performance of the model (*23*). RMSE values were obtained using Eq. 4 with *p*=0.

### Modelling of Salmonella regrowth after treatment with photoactivated chlorophyllin-chitosan complex

Regrowth data were fitted to different models using the DMFit (*24*) shareware package for Excel. The equation of Baranyi and Roberts (*24*) is as follows:
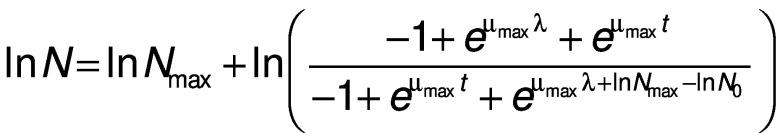
where *N*_0_ and *N*_max_ (measured as absorbance) are the lower and upper asymptotic values and approximately equal to the initial and maximal population density, *μ*_max_ (h^-1^) is the maximum growth rate and *λ* (h) is the latency time.

The Gompertz equation (*25*) states that:
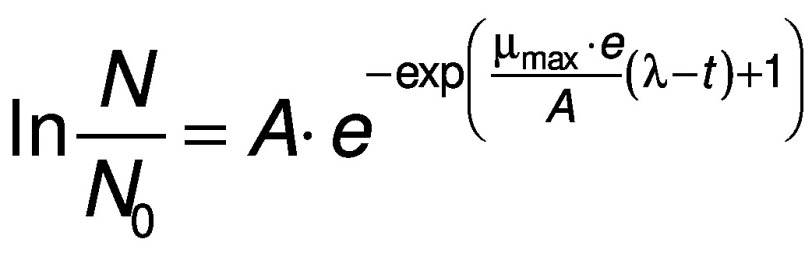
where *A* (absorbance) represents the maximum size of the microbial population.

## RESULTS AND DISCUSSION

### Modelling the inactivation of Salmonella enterica by photoactivated chlorophyllin-chitosan complex

The inactivation of *S. enterica* by a photoactivated chlorophyllin and chitosan complex *in vitro* was investigated using different incubation times (5-60 min) and the potential antimicrobial effects of individual experimental factors were also investigated in order to assess if the observed inactivation requires the combination of these factors.

Ten models were applied for the analysis of microbial inactivation parameters and the results for the three best performing ones are shown in Table 1. Only treatments with photoactivated chlorophyllin and its complex with chitosan caused significant inactivation. The slope of the log-linear model for treatments with chitosan and chlorophyllin-chitosan complex without photoactivation showed that they did not inactivate the pathogen. Comparing the different models for treatment with photoactivated complex, it can be seen that the Weibull model was the one that fitted the best since its RMSE value was the closest to 0 and its R^2^ was the closest to 1 (*20*). In the case of photoactivated chlorophyllin treatment, both Weibull and log-linear+tail models yielded similar results. Therefore, the Weibull model was selected to compare both inactivation curves because it fitted well the two curves. The use of a single model allows comparing kinetic parameters, which are particular for every model and it is only possible when the same model can be applied for different activation curves. The scale parameter for photoactivated complex treatment was four times lower than that for the treatment with photoactivated chlorophyllin, which indicated that this treatment has higher inactivation efficacy. The good fitting of the Weibull distribution to the data is an excellent indicator that the kinetics of *Salmonella* inactivation by photosensitized chlorophyllin-chitosan complex is a consequence of the progressive inactivation of *Salmonella* cells having different photosensitization resistances (*26*). This outcome is not surprising given the nature of our experiments. It should be noticed that in the current case, the microbial inactivation has been described not as a function of photosensitization time but as a function of the time of incubation of *Salmonella* cells with the chlorophyllin-chitosan complex. Therefore, a progress in the inactivation curve should mean more accumulation of the chlorophyllin-chitosan complex on the cell (*14*) and consequently, higher sensibility to light inactivation. This result can be useful for selecting photosensitizing concentrations for further studies of other parameters, such as illumination time. It should be noticed that the validity of this and other models is restricted to the microorganism under study. Other microorganisms can render curves that can be fitted by other models or even by the same model but with other parameter values. Further studies of the effect of this inactivation method on other microorganisms and in real foods would be beneficial for the overall assessment of the efficacy of this method for achieving food safety goals.

**Table 1:** Microbial kinetics modelling

Treatment	Parameter	Model		
Log-linear	Weibull	Log-linear+tail
Photoactivated chlorophyllin-chitosan complex	RMSE R^2^ log*N*_0_/(log CFU/mL) *k*_max_/min^-1^ *δ*/min *p* log*N*_res_/(log CFU/mL)	1.3203 0.6996 4.8±0.4 0.21±0.03	0.3633 0.9773 7.0±0.2 0.04±0.03 0.27±0.02	0.7436 0.9047 6.2±0.3 0.72±0.08 0.7±0.2
Photoactivated chlorophyllin	RMSE R^2^ log *N*_0_/(log CFU/mL) *k*_max_/min^-1^ *δ*/min *p* log*N*_res_	0.1326 0.7578 6.78±0.05 0.03±0.00	0.1297 0.7683 6.83±0.06 106.9±25.6 0.7±0.2	0.1264 0.7797 6.84±0.06 0.05±0.02 6.1±0.2
	RMSE R^2^ log*N*_0_/(log CFU/mL) *k*_max_/min^-1^	0.1271 0.0191 6.77±0.04 0	---	---
chlorophyllin-chitosan complex	RMSE R^2^ log*N*_0_/(log CFU/mL) *k*_max_/min^-1^	0.1438 0.0781 6.82±0.09 0	---	---

RMSE=root mean square error, log*N*_0_=predicted logarithm of the initial count, *k*_max_=specific inactivation rate, *δ*=scale parameter, *p*=shape parameter, log*N*_res_=predicted logarithm of residual count. Estimated parameters are expressed as mean value±standard error

The RMSE index was used as a measure of the performance of the Weibull model to fit the inactivation data (Table 2). RMSE for treatments with photoactivated chlorophyllin and its complex with chitosan were low and close to those found when the models were built, indicating good performance. A point-by-point analysis of the treatment with photoactivated chlorophyllin shows that the model underestimates from the 30 min of treatment on; however, the efficacy of this treatment was poor; therefore, its real-life application is unlikely. Treatment with photoactivated chlorophyllin was indeed useful in the frame of the current research only to test the potential enhancement of lethality if chlorophyllin complexed with chitosan was used. Results of the treatment with photoactivated chlorophyllin-chitosan complex show that the model overestimates the initial population level and the middle part of the inactivation curve, moreover, it underestimates the rest of the curve. The underestimation observed at 60 min of the treatment has no practical relevance since at that time the counts had already fallen to zero. Further studies should validate the model in real food systems.

**Table 2:** Validation of the Weibull model for the inactivation of *S. enterica* in phosphate-buffered saline by photoactivated chlorophyllin and photoactivated chlorophyllin-chitosan complex, *N*=3

		Treatment						
		Photoactivated chlorophyllin				Photoactivated chlorophyllin-chitosan complex		
*t*/min		Observed	Predicted	Predicted - observed		Observed	Predicted	Predicted - observed
0		6.74	6.83	0.09		6.76	6.96	0.20
30		6.72	6.41	-0.31		1.62	0.99	-0.63
45		6.58	6.28	-0.30		0.00	0.29	0.29
60		6.68	6.16	-0.52		0.00	-0.24	-0.24
RMSE				0.3424				0.4065

### Predictive modelling of regrowth of inactivated Salmonella enterica population

**Fig. 2:**
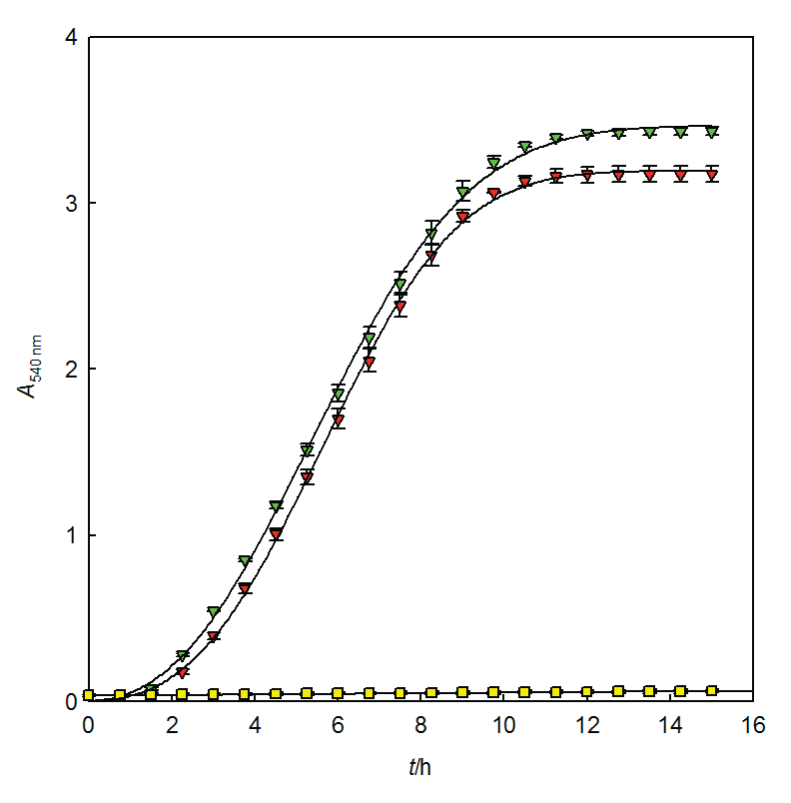
Growth curve of *S. enterica* after inactivation by photoactivated chlorophyllin (red triangle) and chlorophyllin-chitosan complex (green triangle). Control (yellow square)=*S. enterica* without treatment. Curves are fitted to Baranyi and Roberts model (*24*). Bars represent standard deviation. Data from Buchovec *et al.* (*9*, *14*)

While a high level of microbial inactivation can render a food safe and stable, surviving microorganisms can grow during food storage and pose a threat to their safety and stability. This is the reason why it is important to evaluate not only the microbial inactivation but also the regrowth of the surviving population. After the tests of inactivation by photoactivated chlorophyllin-chitosan complex, the dynamics of regrowth of *S. enterica* populations was followed (Fig. 2 (*9*, *14*, *24*)). This included photoactivated chlorophyllin alone and in the complex with chitosan because other treatments did not lead to inactivation. The regrowth of the untreated *S. enterica* population was also evaluated for comparison (control). It can be observed that treatment with photoactivated chlorophyllin-chitosan complex caused damage to *S. enterica* population that made it impossible to regrow during the first 15 h after the treatment under the culturing conditions used in this research. The experiments were not followed beyond 15 h since the growth curves corresponding to the other two treatments reached the stationary phase at that time. In contrast, *S. enterica* population treated with photoactivated chlorophyllin can regrow in a similar way as control bacteria. The absence of regrowth can indicate that either no bacterial cells survived the photosensitization treatment or survivors were sublethally damaged and unable to grow. Sublethal injury has been reported for *Escherichia coli* and *Staphylococcus aureus* cells subjected to photosensitization with curcumin and blue light (*27*).

Two growth models, the Baranyi and Roberts model and the Gompertz model, were tested for fitting regrowth curves. The Baranyi and Roberts model (*24*) yielded a good fit for those populations that were able to grow. The control *Salmonella* grows with a maximal growth rate *μ*=0.46 h^-1^, and a lag phase of 1.69 h (Table 3). When these values were statistically compared with those obtained in the treatment with photoactivated chlorophyllin using the Student’s *t* test, a p=0.2675 was found for the maximal growth rate, therefore, both populations grew at the same rate. In contrast, when the same test was used to compare lag phase duration, a p=0.002 was obtained, which indicated that treatment with photoactivated chlorophyllin causes a post-treatment damage to *S. enterica* strong enough to stop its growth. When the growth parameters of the treatment with photoactivated chlorophyllin-chitosan complex were compared with those of the control, the maximal growth rate of the latter was 291 times higher, indeed, the populations in this treatment did not pass the lag phase. Similar results were reported when *S. enterica* was treated with photosensitized chlorophyllin and the light source was a pulsed light system, which provides high-intensity broad-spectrum light (*28*). However, while pulsed light is known to require short exposure times, it is still an expensive technology.

**Table 3:** Microbial regrowth modelling

Model	Treatment	*µ*/h^-1^	lag phase/h	R^2^
Baranyi and Roberts	Control	0.46±0.02	1.7±0.1	0.9910
	Photoactivated chlorophyllin	0.48±0.02	2.3±0.1	0.9856
	Photoactivated chlorophyllin-chitosan complex	0.002±0.000	15.000	0.0191
				
Gompertz	Control	0.55±0.03	2.31±0.07	0.9920
	Photoactivated chlorophyllin	0.56±0.02	2.8±0.1	0.9838
	Photoactivated chlorophyllin-chitosan complex	0.008±0.004	0.1±0.2	0.0521

Control=no sensitizer and no light. Estimated parameters are expressed as mean±standard error

Determining regrowth potential is important for prediction of the safety of foods after microbial inactivation. The inactivation of foodborne pathogens is seldom complete and is sometimes overestimated when viable but nonculturable state is induced. Therefore, the regrowth potential should be assessed. The absence of regrowth after the treatment with photoactivated chlorophyllin-chitosan complex contrasts with the well-known recovery that bacteria can undergo after UV-C light treatment, both in the dark or under illuminated conditions (*29*). Regrowth has also been observed to occur after the application of other non-thermal methods such as high hydrostatic pressure (*30*) and pulsed light (*31*). Besides the effect of this method on the inactivation of other microorganisms, further studies such as its application to foods and potential effects on food quality and shelf-life are advised.

## CONCLUSION

The inactivation kinetics of Gram-negative food pathogen *Salmonella enterica* by a chlorophyllin-chitosan complex activated with LED-based light at *λ*=405 nm and its regrowth were modelled. Photoactivated chlorophyllin-chitosan complex treatment was able to decrease *S. enterica* counts and no regrowth was observed after 15 h of incubation. Weibull and Baranyi models were the best to describe the inactivation and regrowth kinetics respectively. Validation showed the good performance of the Weibull model to describe the inactivation kinetics. Further studies should validate the models in real food systems. The high inactivation efficacy of this treatment and the lack of recovery of populations afterwards unlocks its huge potential as promising nonthermal and non-chemical approach to control food pathogens on different surfaces.
